# Functional coupling of the lateral prefrontal cortex and the default mode network predicts performance in mental rotation

**DOI:** 10.1162/IMAG.a.112

**Published:** 2025-08-14

**Authors:** Radek Ptak, Naz Doganci, Emilie Marti, Sélim Yahia Coll

**Affiliations:** Research Group Spatial Attention, Perception and Action, Faculty of Medicine, University of Geneva, Geneva, Switzerland; Division of Neurorehabilitation, Department of Clinical Neurosciences, Geneva University Hospitals, Geneva, Switzerland; University Service of Neuropsychology and Neurorehabilitation, Department of Clinical Neuroscience, University Hospitals of Lausanne, Lausanne, Switzerland

**Keywords:** mental rotation, resting-state, functional connectivity, default mode network, motor planning

## Abstract

Mental transformations, such as mental rotation, rely on motor representations and engage neural processes similarly to physical actions. Neuroimaging studies reveal that mental rotation activates the occipito-parietal cortex and motor-related areas, with differences based on whether stimuli are bodily or non-bodily. These findings emphasize the role of frontoparietal networks in mental rotation, similar to those used in motor planning. This study investigated whether resting-state functional connectivity of the left lateral prefrontal cortex (lPFC), a region linked to motor planning, and other functional brain networks predicts mental rotation performance. Fifty-nine healthy individuals underwent functional magnetic resonance imaging (fMRI) to capture resting-state blood oxygenation level dependent (BOLD) activity and completed mental rotation tasks using bodily (hands) and non-bodily (letters) stimuli. Performance in both mental rotation tasks exhibited the expected peak of difficulty with completely inverted stimuli, which require a mental transformation of 180 degrees. At the functional level, mental rotation error rates correlated with lPFC connectivity to the default mode network (DMN). However, this relationship was negative and much stronger for the hands task, indicating that lPFC-DMN interactions were associated with poorer mental rotation performance. These results indicate that effective mental rotation relies on the functional disconnection of the DMN from motor planning networks. The findings highlight the significance of studying resting-state functional connectivity to understand how brain networks contribute to cognitive functions and how their interactions can enhance or impair performance. This work advances our understanding of the neural mechanisms underlying mental rotation, emphasizing the interplay between motor cognition and resting-state dynamics.

## 1 Introduction

Behavioral experiments and functional neuroimaging studies indicate that mental transformations of images, such as those involved in mental rotation, partially depend on motor representations. One key argument is that the mental simulation of motor actions engages similar cognitive processes and neural structures as actual physical actions (Decety & Grezes, 2006; Eaves et al., 2016; Jeannerod, 2001; Munzert et al., 2009; Ptak et al., 2021; Zabicki et al., 2017). A strong alignment is observed between real and imagined actions, whether involving finger movement sequences (Sirigu et al., 1996), object rotation (Gardony et al., 2014), or more complex whole-body activities such as walking uphill or downhill (Courtine et al., 2004). The resemblance between action simulation and execution extends beyond processing speed (Gentili et al., 2004; Sirigu et al., 1996) to encompass other characteristics, such as the perceived effort (Decety et al., 1991) and the spatial dynamics of movement (Papaxanthis et al., 2002).

A second argument is that motor imagery—the mental representation of motor acts—is similarly affected by biomechanical constraints as physical actions. For instance, the time required to judge the laterality of a depicted hand correlates with the time needed for individuals to mentally rotate their own hand to match the stimulus position (Parsons, 1987). Response times are particularly slow for stimuli depicting hand positions that are either difficult to achieve or biomechanically impossible (Overney et al., 2005). Additionally, simultaneous manual object rotation interferes with mental rotation when the two are performed in opposite directions (Wexler et al., 1998; Wohlschlager, 2001; Wohlschlager & Wohlschlager, 1998). Patients with complex regional pain syndrome exhibit slower response times during motor imagery tasks that involve the affected limb (Coslett et al., 2010; Schwoebel et al., 2001). Moreover, mental transformations of non-bodily objects improve when their coordinates are mapped onto one’s own body (Amorim et al., 2006). Collectively, these findings suggest that motor imagery is subject to the same biomechanical constraints as physical actions. Furthermore, they indicate that motor planning plays a causal role in mental rotation involving body parts and, to a lesser extent, non-bodily objects.

A third line of evidence suggests that brain regions critical for motor planning are also engaged during mental rotation of body parts and objects (Cona & Scarpazza, 2019; Kosslyn et al., 1998; Menéndez Granda et al., 2022; Perruchoud et al., 2016; Vingerhoets et al., 2002). An early meta-analysis of neuroimaging studies demonstrated that mental rotation involves widespread activation of the occipito-parietal cortex as well as bilateral motor and premotor cortices (PMC) (Zacks, 2008). These activations include the supplementary motor area (SMA), as well as lateral ventral and dorsal PMC. A study comparing mental rotation with bodily and non-bodily stimuli confirmed similar functional activation patterns but identified distinct differences: bodily stimuli elicited greater activation in the superior parietal cortex bilaterally, the left dorsal PMC, and the SMA, whereas non-bodily stimuli were associated with increased activation in the right inferior parietal and superior occipital regions (Tomasino & Gremese, 2015).

Although fMRI BOLD patterns cannot confirm causality in brain functions, more direct evidence for the contribution of motor areas to mental rotation comes from transcranial magnetic stimulation (TMS) and lesion studies. TMS applied to the left primary motor cortex disrupted mental rotation of hands but had no effect on mental rotation of letters (Tomasino et al., 2005). Similarly, inhibitory TMS to the posterior parietal cortex impaired mental rotation performance in visuomotor rotation tasks (Bestmann et al., 2002). More recently, Cona et al. (2017) showed that TMS targeting the dorsal PMC specifically impaired mental rotation. A meta-analysis further revealed that TMS to frontal or parietal regions disproportionately affects mental rotation of bodily stimuli compared to objects (Veldema et al., 2021). Lesion studies also support these findings. Small-scale studies have linked mental rotation impairments to parietal cortex damage, with some emphasizing the right hemisphere (Ditunno & Mann, 1990; Ratcliff, 1979), others implicating the left hemisphere (Carota et al., 2004; Mayer et al., 1999), and some suggesting a dissociated pattern (Tomasino & Rumiati, 2004; Tomasino et al., 2003). A large-scale lesion study comparing mental rotation deficits for bodily and non-bodily stimuli found that damage to the left primary motor, somatosensory and superior parietal cortices impaired mental rotation for both stimulus types, while deficits specific to non-bodily objects were additionally linked to inferior parietal lobe damage (Doganci et al., 2024).

**Fig. 1.**
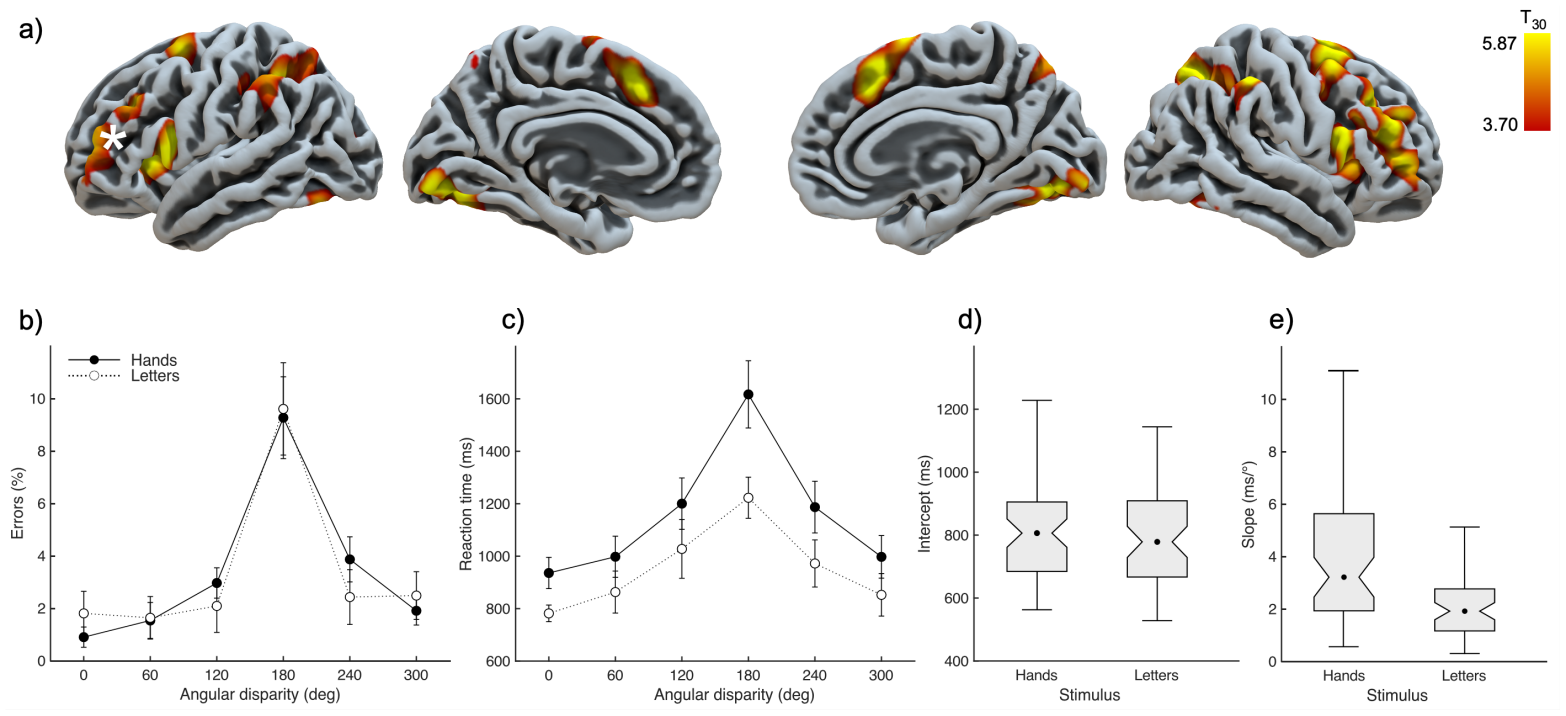
Seed selection and results of mental rotation tasks. (a) Contrast of functional activations between self-selected versus visually instructed finger movements. Active-state FC analysis revealed a bilateral frontoparietal network connected to the left lPFC, which was consequently chosen as seed in the current study (white star). Data from Doganci et al. (2023b). (b) Percent errors and (c) reaction times in the hand and letter rotation task, as a function of angular disparity. (d) Intercept and (e) slope of reaction times in the hand and letter rotation task.

This evidence from neuroimaging, neurostimulation, and lesion studies highlights the critical role of frontoparietal networks in mental rotation. Interestingly, these networks are also recruited during the planning and execution of reaching and grasping movements (Begliomini et al., 2014; Filimon, 2010; Ptak et al., 2017). In previous work, we found that the left lateral prefrontal cortex (lPFC) is part of a network engaged in motor planning (Doganci et al., 2023b). The network was identified based on active-state functional connectivity (FC) during a simple motor planning task and consisted of bilateral dorsal frontoparietal cortex and the left lPFC (Fig. 1). While previous studies have primarily relied on activation measures, it remains unclear whether mental rotation performance can be predicted from resting-state FC. Resting-state fMRI has a distinct advantage over task-induced fMRI activations in identifying brain networks based on the correlation of hemodynamic activity, which is interpreted as ‘collaboration’ between network regions. Large-scale analyses of resting-state data suggest that, even at rest, the brain is organized into several large-scale networks (van den Heuvel & Hulshoff Pol, 2010; Yeo et al., 2011). Investigating how these networks contribute to specific cognitive functions offers valuable insights into the structure and specialization of cortical interactions underlying behavior. Of particular interest is the interplay between ‘task-positive’ networks—those that become active during tasks, such as the motor network—and ‘task-negative’ networks like the default mode network (DMN), which show heightened activity during rest or when no task is being performed.

Building on the established link between motor planning and mental rotation, we hypothesized that resting-state FC between the lPFC and brain regions that are relevant for the processing and transformation of mental representations is associated with mental rotation performance. Since we hypothesize that mental rotation relies on how motor processes integrate with mental transformations, we expected this association to be positive. To address this, we assessed healthy participants on two mental rotation tasks—one involving bodily stimuli (hands) and another using non-bodily stimuli (letters)—and analyzed correlations between FC and task performance. By focusing on these relationships, this study sought to describe the resting-state networks associated with mental rotation.

## 2 Materials and Methods

### 2.1 Participants

Sixty-one healthy, right-handed participants were recruited for this study through flyers distributed at the University of Geneva and the University Hospitals of Geneva. None had a history of neurological or psychiatric disorders. Based on a previous study including 24 participants (Doganci et al., 2023a) who performed the hand and letter mental rotation tasks (see description below), we computed that a sample size of 52 was required to identify a significant difference between the rotation effects in both tasks (reaction time measured at 180 compared to 0 degrees; false positive rate = 0.05; power = 80%).

Two participants were excluded from the analyses due to their unavailability for behavioral assessment. Consequently, the final sample comprised 59 healthy, right-handed individuals (mean age = 49.2 ± 19.4 years; 27 females) who successfully completed both behavioral and functional experiments and were included in the analyses. Behavioral testing and imaging were conducted in two separate sessions, spaced apart by an average of 4 ± 57.6 days. All participants provided written informed consent before participating. The study was approved by the Ethical Commission of the Canton of Geneva, Switzerland, and adhered to the principles outlined in the Declaration of Helsinki.

### 2.2 Mental rotation tasks

Participants performed two mental rotation tasks, conceptually similar but differing in the type of rotating stimulus (hand vs. letter; for a detailed description, see Doganci et al., 2023a). Tasks were programmed using E-Prime 3.0 (Psychological Software Tools Inc., Pittsburgh, PA). For the hand task, black-and-white photographs of a right male hand in a palm-down view (12.3 × 9.2 degrees) were used. The images depicted either the right hand or its mirror-reversed version representing the left hand. In the letter task, five asymmetrical capital letters (L, F, G, P, R) in black Arial font (8.4 × 7.1 degrees) were displayed in either their canonical or mirror-reversed versions. Stimuli for both tasks were shown at six angular disparities: 0°, 60°, 120°, 180°, 240°, and 300°. The hand task consisted of 192 trials presented across four blocks of 48 trials (32 trials per condition). The letter task included 180 trials divided into three blocks of 60 trials (30 trials per condition). Experimental conditions in each block were orthogonalized and presented in randomized order. Stimuli were presented centrally on a 13.3-inch laptop (Hewlett Packard Elite X360) positioned 50 cm from the participant, and verbal responses were registered with an external microphone connected to the laptop. The microphone was attached to a headset and positioned close to the participant’s mouth. This setup ensured high-quality recordings and helped avoiding invalid responses.

Participants were instructed to rest their hands on their lap and to avoid any hand movement during the experiment. In the hand task, they responded verbally by saying “left” or “right” to indicate the parity of the stimulus. In the letter task, participants responded with “correct” or “incorrect” to specify whether the letter was in its canonical or mirror-reversed form. Verbal responses were chosen over manual ones to minimize interference with body imagery processes, as suggested by Cocksworth and Punt (2013). Once a response was given, the experimenter pressed either the left or right arrow key to log it. This allowed later identification of the response type (left arrow: “left hand” or “incorrect”; right arrow: “right hand” or “correct”). Participants completed 15 practice trials for each of the two tasks. During these trials, their responses were monitored, and reminders were provided when necessary to ensure they used only the specified response alternatives. Vocal responses were recorded for up to 10 seconds and later analyzed offline using a custom MATLAB script, which enabled playback and manual marking of the response onset.

### 2.3 Functional imaging

#### 2.3.1 fMRI acquisition and pre-processing

Structural and functional magnetic resonance imaging, including a resting-state fMRI (rs-fMRI) scan, was conducted in a single session. Scanning was performed on a 3T Trio scanner equipped with a 64-channel array coil (Siemens Medical Solutions, Erlangen, Germany). High-resolution structural images were acquired using T1-weighted MPRAGE sequences (TR = 2,300 ms; TE = 1.96 ms; FA = 9°; voxel size = 1.0 mm isotropic; 176 slices). Functional images were obtained using a fast echoplanar imaging (EPI) sequence (TR = 720 ms; TE = 30 ms; FA = 50°; voxel size = 2.5 mm isotropic; 56 axial slices; 440 volumes). During image acquisition, participants lay in the scanner with their heads stabilized using cushions to minimize movement. They were instructed to keep their eyes open and focus on a centered white fixation cross displayed on a black background in a darkened environment.

Anatomical and functional data were preprocessed using a flexible pipeline implemented in the CONN toolbox (Whitfield-Gabrieli & Nieto-Castanon, 2012). Functional data were realigned, with all scans coregistered to a reference image and resampled to correct for motion and magnetic susceptibility interactions. Temporal misalignment between slices was addressed using sinc temporal interpolation, aligning each slice’s BOLD time series to a common mid-acquisition time. Outlier scans (i.e., scans with a framewise displacement above 0.9 mm) were identified with the Artifact Detection Tools (ART; Power et al., 2014), and a reference BOLD image was created for each participant by averaging all scans excluding outliers. We accepted a maximum of 10% outlier scans as a criterion to include a participant in the analysis, which was met by all 59 participants.

Normalization of anatomical and functional data into standard MNI space with resolutions of 1 mm (structural) and 2 mm (functional) isotropic voxels was performed using the unified segmentation and normalization algorithm in SPM12 (http://www.fil.ion.ucl.ac.uk/spm). Subsequently, spatial smoothing was applied using an 8-mm full-width-at-half-maximum (FWHM) Gaussian kernel. Denoising followed a standard pipeline, regressing out potential confounds, including white matter and cerebrospinal fluid (CSF) time series, 12 potential noise components defined from the subject-motion parameters, outlier scans, session effects, and linear trends within each functional run. Bandpass filtering was applied to the BOLD time series, retaining frequencies between 0.008 Hz and 0.09 Hz. Noise components derived from white matter and CSF regions were estimated (Behzadi et al., 2007), contributing to the effective degrees of freedom in the denoised BOLD signal.

#### 2.3.2 ROI-based and seed-based FC analyses

To test whether mental rotation is associated with FC between the lPFC and resting-state networks, we conducted region-of-interest (ROI) and seed-to-voxel analyses. ROI-to-ROI analyses were performed to investigate whether distinct networks could be identified in our sample based on baseline resting-state functional connectivity (FC). These analyses focused on connectivity between multiple ROIs derived from the Harvard-Oxford (H-O) atlas. ROI selection was informed by the activation foci identified in a previous fMRI study of motor planning (Doganci et al., 2023b). This study contrasted brain activations during self-initiated finger movements with those triggered by external cues. The contrast isolated a cognitive component specific to selecting a motor action with a particular effector, resulting in 16 activation clusters across the bilateral fronto-parietal and occipito-temporal cortices (Fig. 1a). From the H-O atlas, we selected ROIs corresponding to the locations of these clusters, including the superior frontal gyrus (SFG), middle frontal gyrus (MFG), opercular part of the inferior frontal gyrus (IFG), superior parietal lobe (SPL), inferior parietal lobule (IPL; corresponding to the angular gyrus in the H-O atlas), fusiform gyrus, and lingual gyrus. To evaluate ROI-to-ROI connectivity, pairwise comparisons were calculated across the regions defined by the H-O atlas, yielding a total of 136 connection pairs. Functional connectivity (FC) networks were identified through hierarchical clustering of these ROI-to-ROI connections, applying a cluster-level false-discovery rate (FDR) correction of *p* < 0.05.

Unlike ROI-to-ROI connectivity, which focuses on predefined brain regions, seed-to-voxel connectivity examines all voxels across the entire brain to identify those that show significant FC with a specified seed region. In our previous functional imaging study, the lPFC was found to form a functional network with bilateral fronto-parietal cortices, implicated in motor planning (Doganci et al., 2023b). The lPFC plays a key role in behavioral organization, regulation, and control, contributing to motor planning by selecting action plans and overseeing their execution (Friedman & Robbins, 2022; Miller & Cohen, 2001; Szczepanski & Knight, 2014). Based on these findings, the lPFC was chosen as the seed region for the seed-to-voxel analysis (Fig. 1a), defined using MNI coordinates derived from the lPFC activation cluster revealed in our previous motor planning study (Doganci et al., 2023b). The seed was defined as a sphere with 10 mm diameter, centered at the MNI-coordinates x = -30, y = 48, z = 18, which corresponds to the left inferior middle frontal gyrus (MFG). The seed-to-voxel connectivity analysis thus identified all brain regions whose connectivity with the left MFG significantly predicted performance in the mental rotation tasks. To derive meaningful performance indices from the two mental rotation tasks, we computed for each task the difference between RT and error rates at the 180° and the 0° condition. Since mental rotation is the most difficult at 180° (see Fig. 1), this difference score reflects maximal rotation (180°) compared to no rotation (0°).

Functional connectivity strength was measured using Fisher-transformed bivariate correlation coefficients, computed within a weighted general linear model (GLM; Nieto-Castanon, 2020). Group-level analyses employed a GLM framework, and voxel-level hypotheses were assessed using multivariate parametric statistics. Cluster-level inferences were made using Gaussian Random Field theory, with a voxel-level threshold of *p* < 0.001 and a cluster-level familywise error (FWE)-corrected threshold of *p* < 0.05.

## 3 Results

### 3.1 Behavioral results

Figure 1 shows behavioral results of the hand and letter mental rotation tasks. A repeated-measures ANOVA with the factors stimulus (hand, letter) and angular disparity (0°, 60°, 120°, 180°, 240°, 300°) was performed on the error data and RTs. Where necessary, a Huynh-Feldt correction of the degrees of freedom was applied to compensate for violations of sphericity.

The analysis of error data (Fig. 1b) yielded a significant effect of angular disparity (*F*_1.69,58_ = 32.16, *p* < 0.001), while the factor stimulus and the interaction were not significant (*p* > 0.36). In contrast, the analysis of RT data (Fig. 1c) revealed a significant effect of stimulus (*F*_1,58_ = 5.41, *p* < 0.05), angular disparity (*F*_1.95,58_ = 42.53, *p* < 0.001), and the interaction (*F*_1.93,58_ = 5.95, *p* < 0.01). We did not follow up the interaction since only the comparison between performance at 0° (no rotation) and 180° (max. rotation) is of relevance for this study. However, we computed with the RT data of each participant a linear regression across the first four angular disparities (0°, 60°, 120°, and 180°) and thus determined the individual intercept (Fig. 1d) and slope (i.e., increase of RT per 1° of angle; Fig. 1e). T-tests comparing these values showed a significantly higher slope for hands than letters (*t*_58_ = 4.34, *p* < 0.001), but a comparable intercept (*t*_58_ = 0.48, *p* = 0.63).

Thus, RT data indicated that subjects found the hand rotation task more challenging as expressed in increased RTs and a larger slope of the regression. However, this did not translate into a larger number of errors.

### 3.2 ROI-to-ROI connectivity

**Fig. 2.**
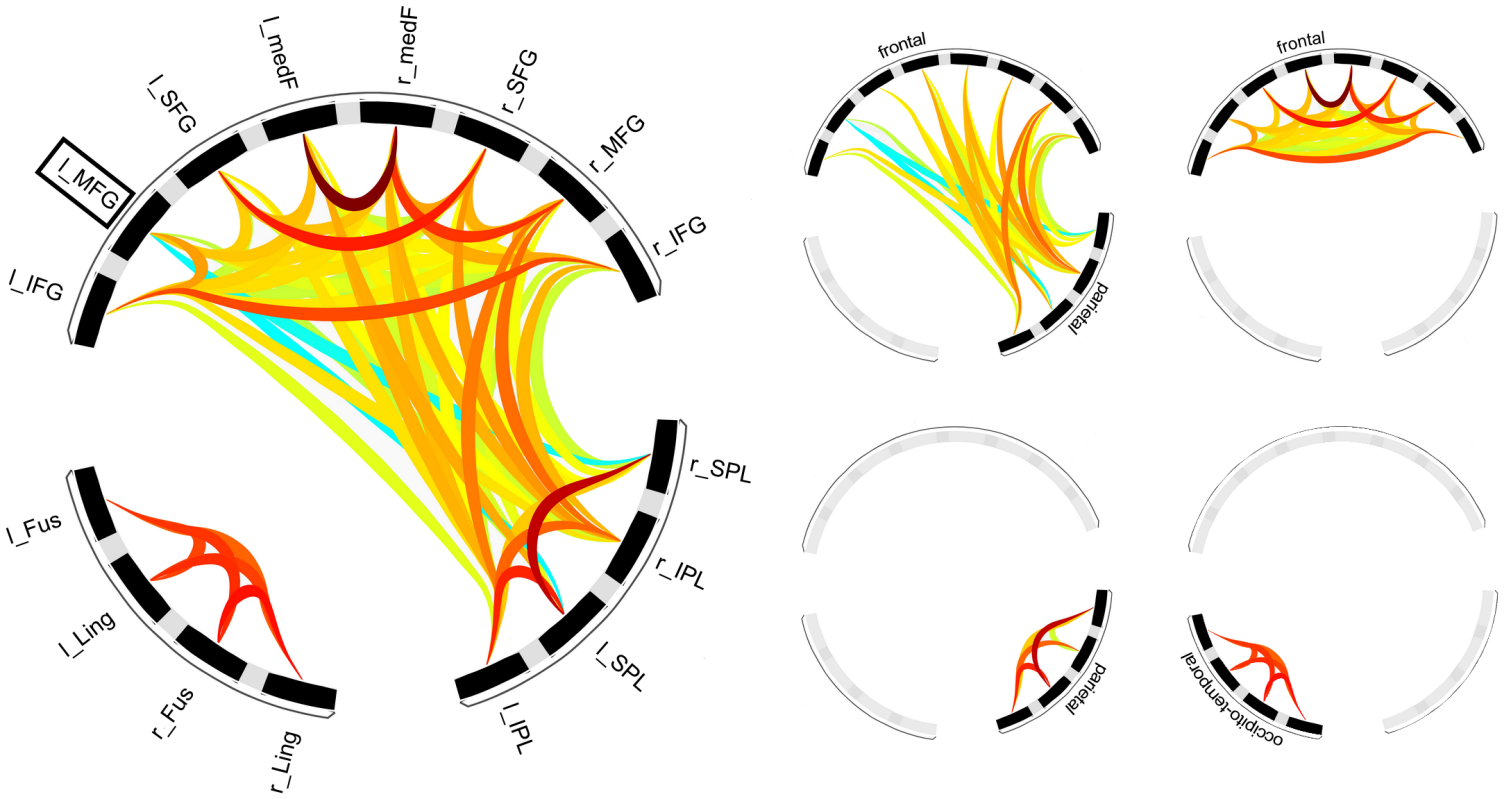
Results of the ROI-to-ROI connectivity analysis. All significant connections among the 16 regions of interest are shown on the left. The right side shows connections of four clusters separated through hierarchical clustering (Abbreviations: Fus: fusiform gyrus; IFG: inferior frontal gyrus; IPL: inferior parietal lobe; Ling: lingual gyrus; medF: medial frontal cortex; MFG: middle frontal gyrus; SFG: superior frontal gyrus; SPL: superior parietal lobe). The l_MFG corresponds to the seed area used in the seed-to-voxel analysis.

An ROI-to-ROI connectivity analysis was conducted to examine the resting-state networks constituted by 16 brain regions involved in motor planning (Doganci et al., 2023b). Similar to an investigation of active-state FC in the latter study, the analysis identified two segregated networks, respectively consisting of 12 frontoparietal regions and four occipito-temporal regions (Fig. 2; Supplementary Table). Hierarchical clustering further subdivided the frontoparietal network into three sub-networks consisting of intra-frontal, intra-parietal and frontoparietal connections, respectively. Of note, the atlas region that contained the lPFC seed (i.e., the left MFG) exhibited positive FC at rest with all left frontal regions and the right SPL, as well as negative FC with all parietal regions except the left IPL.

### 3.3 Seed-to-voxel connectivity

**Fig. 3.**
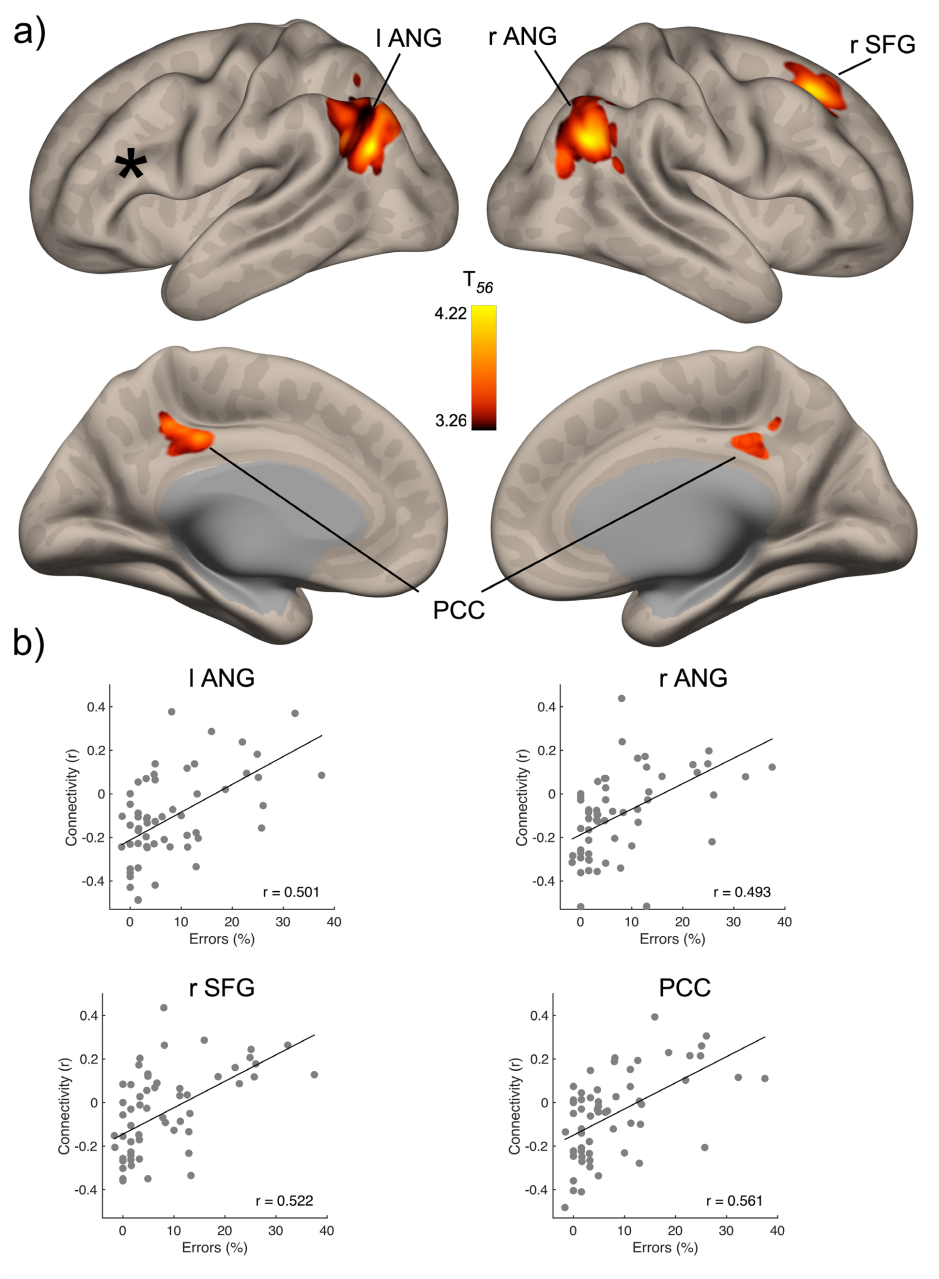
Results of global seed-based connectivity analyses. (a) Clusters in which functional connectivity with the seed (black star) correlated significantly with error rates across both mental rotation tasks. (b) Scatterplots showing the correlation between connectivity averaged across all voxels within a cluster and error rates. ANG: angular gyrus; PCC: posterior cingulate cortex; SFG: superior frontal gyrus.

Seed-based regression analyses aimed to identify all voxels whose connectivity with the lMFG seed significantly correlated with behavioral performance. In a first analysis, we examined whether FC was associated with mental rotation performance irrespective of the stimulus (hand or letter). The analysis using RT yielded no significant anatomical clusters. In contrast, the analysis of error rates identified four significant clusters (Fig. 3a): the left (cluster size: 187 voxels; MNI-coordinates: -52, -70, +32) and right angular gyrus (304 voxels; +54, -62, +34), bilateral posterior cingulate cortex (159 voxels; -06, -32, +36), and the right SFG (161 voxels; +24, +28, +44). FC of all four clusters correlated positively with errors (Fig. 3b), indicating that higher mental rotation performance was associated with lower connectivity.

**Fig. 4.**
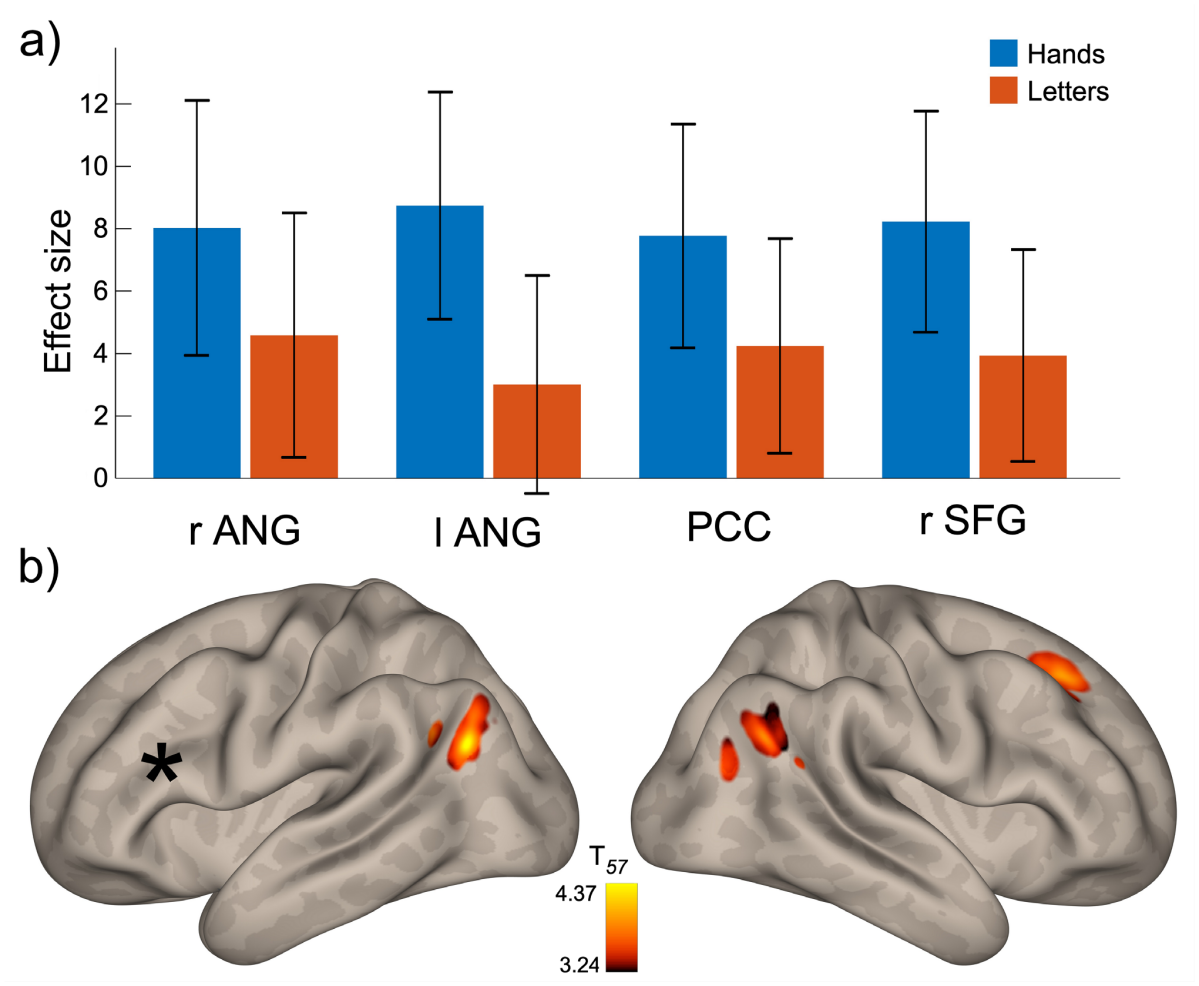
Results of seed-based connectivity analyses. (a) Effect sizes of the seed-based analysis performed across both mental rotation tasks. Values show the adjusted means of connectivity within each cluster for the hands and the letters condition. (b) Clusters in which functional connectivity with the seed (black star) correlated significantly with error rates in the hand task only. ANG: angular gyrus; PCC: posterior cingulate cortex; SFG: superior frontal gyrus.

Figure 4a shows that the effect sizes of the significant FC-performance associations were consistently higher for the hand task than the letter task. To examine whether these associations were driven by both tasks, we additionally conducted separate analyses. Analysis of the hand task identified three significant clusters in the left (266 voxels; -54, -70, +30) and right angular gyrus (253 voxels; +54, -64, +32), as well as the right SFG (174 voxels; +24, +30, +40; Fig. 4b). Again, all FC-behavior correlations were positive. Thus, except for the absence of a significant posterior cingulate cluster, the results were very similar to the global analysis. In contrast, analysis of the letter task did not yield any significant results.

## 4 Discussion

Our study demonstrates that performance in mental rotation, as measured by error rates, is functionally linked to a resting-state network comprising the left ventrolateral prefrontal cortex (lPFC), the left and right inferior parietal lobules (IPL), the posterior cingulate cortex (PCC), and the right superior frontal gyrus (SFG). As expected, participants exhibited a characteristic increase in both error rates and reaction times (RTs) with greater angular disparity, peaking at 180°. This well-established pattern was first reported in the seminal study by Shepard and Metzler (1971), and has been extensively replicated since (Searle & Hamm, 2017). Notably, reaction times in the hands task were longer compared to the letter task, suggesting that bodily processes involved in mental rotation impose additional cognitive demands. This distinction is particularly significant given our finding that resting-state functional connectivity (FC) was associated with the hands task but not with the letter task.

We selected the lPFC as the seed region for brain-wide FC analysis due to its recognized role in motor selection and planning. In a prior functional imaging study, we observed that this region was activated when participants selected one of four fingers to respond, in contrast to a condition where the effector was visually indicated, and no selection was required (Doganci et al., 2023b). Since movement-related activations were identical across conditions, this suggests that the lPFC activity is not linked to movement execution. Instead, these findings indicate that the lPFC is part of a network responsible for conscious effector selection, as well as the preparation and supervision of movements, rather than their execution (Friedman & Robbins, 2022). Based on this evidence implicating the lPFC in motor cognition, we selected it as the seed region for our analysis.

The seed-to-voxel analyses identified the left and right angular gyrus, the PCC, and the right SFG as predictors of mental rotation. These findings do not align with previous meta-analyses of neuroimaging data related to mental rotation. Zacks (2008), who examined 32 studies, reported that mental rotation was associated with activations in both superior parietal lobes, the SFG, and the left lateral frontal cortex. Similarly, based on 60 studies included in their meta-analysis, Tomasino and Gremese (2015) identified a mental rotation network comprising the bilateral superior parietal lobes, the left precentral gyrus, bilateral inferior and middle frontal gyri, the supplementary motor area (SMA), bilateral occipital gyri, and bilateral cerebellum. The most recent meta-analysis by Hiew et al. (2023) confirmed these previous findings. Interestingly, only the SFG appears to be involved both, in brain activation studies, and our resting-state FC study. Its involvement has been hypothesized to reflect strategic components in mental rotation, such as to adopt a visual rather than a motor strategy (Tomasino & Gremese, 2015). In contrast, the other brain regions identified in our seed-to-voxel analysis do not overlap with the mental rotation network revealed in activation studies. Specifically, the involvement of the angular gyri, PCC and SFG is more consistent with a role of the default mode network (DMN) in mental rotation (Laird et al., 2009; Raichle et al., 2001). The DMN is characterized by decreased fMRI BOLD signal during task performance and increased activation during states of rest (Mantini & Vanduffel, 2013). Notably, DMN activity is anticorrelated with that of other brain networks, particularly attention networks, which are typically recruited during externally driven tasks (Fox et al., 2005; Ptak, 2012; Shulman et al., 1997). The recruitment of the DMN during rest has been interpreted as evidence that much of cognitive processing relies on intrinsic, inwardly directed activity (Raichle, 2010). Thus, ‘rest’ does not signify an absence of processing but rather the presence of sustained, internally focused functional activity.

In a typical mental rotation task, the target image is presented visually, after which participants perform a cognitive transformation of mental images. This involves mentally rotating a visual representation of the target through the shortest angle to align it with an internal template (Searle & Hamm, 2017; Shepard & Metzler, 1971). Although this is an active cognitive process triggered by external stimulation, it bears resemblance to internal mentation and mind-wandering, which have been proposed as core functions of the DMN (Mantini & Vanduffel, 2013). Importantly, unlike task-induced activations, predicting behavioral performance based on resting-state functional connectivity (FC) highlights fundamental traits of the studied brain regions rather than state-dependent characteristics (Bernstein-Eliav & Tavor, 2024). Interpreting the involvement of the DMN in our study hinges, therefore, on the possible similarity between the core functions of this network and its trait-dependent features revealed by the correlation between FC and mental rotation performance. However, since behavioral performance was indexed by error rates, the observed positive correlation between FC and performance suggests that greater intrinsic FC between the lPFC and the DMN was associated with poorer participant performance. In other words, better mental rotation performance was linked to a *reduction* in FC.

While interpreting correlation results can be challenging, this finding implies that trait-dependent features of the DMN do not enhance mental rotation performance. Meta-analyses of fMRI activity during mental rotation tasks consistently report activations in superior parietal, occipital, and premotor regions—areas that lie outside the DMN (Hiew et al., 2023; Ptak et al., 2017; Tomasino & Gremese, 2015; Zacks, 2008). Previous studies examining the association between resting-state FC and behavioral performance on diverse tasks mostly observed positive relationships, both in healthy and pathological populations (Carter et al., 2010; Cole et al., 2013; Fellrath et al., 2016; Marti et al., 2025; Ptak et al., 2020; Ramsey et al., 2016; van den Heuvel & Hulshoff Pol, 2010). Buckner et al. (2013) suggest that resting-state connectivity reflects a state of neural activity that is characterized by specific cognitive operations which might not be involved in a similar way during a task. Meta-analytic findings on brain activation during mental rotation tasks suggest that better performance is associated with reduced involvement of the DMN. In line with this, the current study found a negative relationship between lPFC-DMN connectivity and task performance. This aligns with previous research indicating that the DMN is typically anticorrelated with networks responsible for externally focused visual processing and attention (Fox et al., 2005; Raichle et al., 2001). Nonetheless, given the still-unclear relationship between brain activation and connectivity, it remains uncertain whether DMN-related processes—such as internal mentation and mind-wandering—are simply unnecessary or potentially even disruptive to effective mental rotation.

## 5 Limitations and Conclusions

At the outset of our study, we hypothesized a link between mental rotation and motor cognition. This hypothesis was based on the similarity between motor imagery, action observation, and physical action, as well as the overlap between functional activations associated with mental rotation and those associated with action execution (Cona & Scarpazza, 2019; Eaves et al., 2016; Gentili et al., 2004; Hardwick et al., 2018; Hetu et al., 2013; Munzert et al., 2009; Ptak et al., 2021). We selected the lPFC as seed because this region forms a network with dorsal frontoparietal cortex during action selection (Doganci et al., 2023b), which we expected would also be associated with mental rotation. The lPFC is involved in fronto-parietal circuits activated during the planning and execution of motor commands, and is believed to code the selection and goals of upcoming actions (Filimon, 2010). Identifying a resting-state network that is predictive of mental rotation performance would, therefore, support the view that embodied motor processes are necessary for this cognitive function.

The present data confirm our hypotheses, though only indirectly. First, as anticipated, the lPFC seed is, indeed, implicated in mental rotation, as its network connections are predictive of mental rotation performance. Unexpectedly, we only identified a network that is *negatively* correlated with performance. Given that better mental rotation performance is linked to reduced connectivity between the lPFC and the DMN, this finding suggests that efficient performance may depend on the functional decoupling of the DMN from the motor planning network. However, given the ambiguous relationship between functional connectivity and activation, the significance of this finding—especially concerning the DMN’s role in mental rotation—should be interpreted with caution. In particular, the observed decoupling between two brain regions does not necessarily indicate that either region is irrelevant to the task at hand.

Further, the predictability of behavioral performance from FC was significantly stronger for the hand task than the letter task. This finding aligns with a previous study on the predictability of mental rotation from focal brain activations (Doganci et al., 2023a), which found that activations within several brain regions involved in action selection correlated positively with mental rotation of hands, but not letters. Previous functional imaging studies reported a partial overlap between activations associated with mental rotation of bodily and non-bodily stimuli, whereby both implicated bilateral parietal cortex and motor and premotor cortices (Tomasino & Gremese, 2015). Similarly, voxel-based lesion symptom mapping identified common involvement of left superior parietal, somatosensory, and motor cortex in mental rotation of hands and letters (Doganci et al., 2024). However, despite these similarities, bodily stimuli appear to rely more on brain regions involved in motor planning and execution than non-bodily stimuli.

Given that it is based on a correlational approach, the use of resting-state FC as predictor may identify brain networks that are positively or negatively associated with performance in a specific task. Despite this limitation, our study demonstrates the utility of FC analyses in identifying functional networks that contribute to a specific cognitive function. These networks can either impair performance or enhance performance depending on their connectivity behavior, providing valuable insights into the functional balance between brain networks and their contribution to behavior. Future studies examining the relationship between resting-state networks and task-induced activations will be necessary to establish the overlap between embodied and non-embodied mental rotation.

## Data and code Availability

The raw data supporting the conclusions of this article will be made available by the authors upon reasonable request, without undue reservation.

## Supplementary Material

Supplementary Material
