# Supplementary material for: Functional coupling of the lateral prefrontal cortex and the default mode network predicts performance in mental rotation
Source: Imaging Neurosci (Camb). 2025 Aug 14;3:IMAG.a.112. doi: 10.1162/IMAG.a.112 (PMC12358948; doi:10.1162/IMAG.a.112)
Supplement: Supplementary Material [file IMAG.a.112_supp.pdf]

Supplementary table: Statistical results of the ROI-to-ROI analysis. Numbers written in bold script show statistical results for each of the four clusters.

Cluster	Connection	Statistic	p-FWE
**fronto-parietal**		**F(2,57)=217.99**	**<0.000001**
	r_IPL-r_MFG	t(58)=8.82	<0.000001
	r_MFG-l_IPL	t(58)=8.29	<0.000001
	r_SFG-r_IPL	t(58)=7.47	<0.000001
	l_medF-l_SPL	t(58)=6.8	<0.000001
	r_IPL-r_medF	t(58)=6.6	<0.000001
	l_medF-l_IPL	t(58)=6.57	<0.000001
	r_IFG-r_IPL	t(58)=6.37	<0.000001
	r_medF-l_IPL	t(58)=6.22	<0.000001
	l_SFG-l_IPL	t(58)=5.02	0.000016
	l_IFG-r_IPL	t(58)=5	0.000017
	l_medF-r_SPL	t(58)=4.5	0.00007
	l_SFG-r_IPL	t(58)=4.14	0.000246
	r_medF-r_SPL	t(58)=4.09	0.000202
	r_SFG-l_IPL	t(58)=4.11	0.000272
	l_MFG-l_SPL	t(58)=-4.12	0.000304
	r_MFG-r_SPL	t(58)=3.87	0.00047
	l_MFG-r_SPL	t(58)=-3.55	0.001646
	r_MFG-l_SPL	t(58)=3.42	0.001745
	l_IFG-l_IPL	t(58)=3.21	0.004701
	r_medF-l_SPL	t(58)=2.98	0.005695
	r_IPL-l_medF	t(58)=2.84	0.011524
	r_IFG-l_IPL	t(58)=2.65	0.019601
	r_IFG-r_SPL	t(58)=2.53	0.02357
	l_SFG-l_SPL	t(58)=2.33	0.034968
**Frontal**		**F(2,57)=64.48**	**<0.000001**
	r_SFG-l_MFG	t(58)=16.42	<0.000001
	r_SFG-l_SFG	t(58)=11.47	<0.000001
	r_MFG-r_medF	t(58)=10.65	<0.000001
	l_IFG-r_IFG	t(58)=9.96	<0.000001
	r_SFG-r_medF	t(58)=7.11	<0.000001
	l_MFG-l_IFG	t(58)=7.17	<0.000001
	r_IFG-r_medF	t(58)=6.8	<0.000001
	r_SFG-r_MFG	t(58)=6.59	<0.000001
	r_IFG-r_MFG	t(58)=6.47	<0.000001
	l_SFG-l_medF	t(58)=6.13	0.000001
	l_SFG-r_medF	t(58)=6.04	0.000001
	l_MFG-l_medF	t(58)=5.85	0.000002

	l_SFG-l_MFG	t(58)=5.66	0.000002
	l_IFG-l_medF	t(58)=5.46	0.000005
	l_MFG-r_medF	t(58)=5.25	0.000009
	l_IFG-r_medF	t(58)=5.13	0.000013
	r_SFG-l_MFG	t(58)=5.03	0.000015
	r_SFG-r_IFG	t(58)=4.92	0.000019
	l_IFG-r_MFG	t(58)=4.73	0.000036
	r_MFG-l_medF	t(58)=4.47	0.00007
	l_SFG-r_MFG	t(58)=4.52	0.000076
	r_IFG-l_medF	t(58)=3.78	0.000924
	l_MFG-r_MFG	t(58)=3.26	0.003529
	l_SFG-r_IFG	t(58)=2.66	0.019009
	r_SFG-l_medF	t(58)=2.43	0.026888
	r_SFG-l_IFG	t(58)=2.4	0.026888
	l_SFG-l_IFG	t(58)=2.37	0.034926
**Parietal**		**F(2,57)=153**	**<0.000001**
	r_SPL-l_SPL	t(58)=14.25	<0.000001
	l_IPL-l_SPL	t(58)=11.13	<0.000001
	r_IPL-l_IPL	t(58)=9.09	<0.000001
	l_IPL-r_SPL	t(58)=5.71	0.000001
	r_IPL-r_SPL	t(58)=2.58	0.020487
**Occipito-temporal**		**F(2,57)=69.35**	**<0.000001**
	r_Fus-r_Ling	t(58)=11.84	<0.000001
	l_Ling-r_Fus	t(58)=10.95	<0.000001
	l_Fus-r_Fus	t(58)=10.64	<0.000001
	l_Ling-r_Ling	t(58)=10.3	<0.000001
	l_Fus-l_Ling	t(58)=10.18	<0.000001
	l_Fus-r_Ling	t(58)=9.15	<0.000001
